# Systematic review with meta-analysis: Prevalence, risk factors, and challenges for urinary schistosomiasis in children (USC)

**DOI:** 10.1371/journal.pone.0285533

**Published:** 2023-08-17

**Authors:** Noor Azreen Masdor, Thinakaran Kandayah, Norizzati Amsah, Rahayu Othman, Mohd Rohaizat Hassan, Syed Sharizman Syed Abdul Rahim, Mohammad Saffree Jeffree, Khamisah Awang Lukman, Aizuddin Hidrus

**Affiliations:** 1 Faculty of Medicine, Department of Public Health Medicine, Universiti Kebangsaan Malaysia, Cheras, Kuala Lumpur, Malaysia; 2 Borneo Medical and Health Research Centre, Universiti Malaysia Sabah, Kota Kinabalu, Sabah, Malaysia; 3 Department of Public Health Medicine, Universiti Malaysia Sabah, Kota Kinabalu, Sabah, Malaysia; University of Uyo, NIGERIA

## Abstract

**Background:**

Schistosomiasis is a parasitic infection that causes significant public health problems in tropical countries. *Schistosoma haematobium* species are blamable for causing urinary schistosomiasis. The infected person, specifically children, may be carrying the disease. This systematic review aimed to identify the current knowledge of urinary Schistosmiasis in children or USC on its epidemiology, risk factors, and challenges to spread the understanding of controlling the disease and reducing the complications.

**Method:**

In November 2021, a systematic computer-aided literature review was conducted using PubMed, SCOPUS and Web of Science, following the Preferred Reporting Items for Systematic Reviews and Meta-Analyses (PRISMA) criteria. The results were updated in February 2022. We only used papers that have at least the abstract available in English. Relevant articles were screened, duplicates were deleted, eligibility criteria were applied, and studies that met the criteria were reviewed. The keywords Human Schistosoma infections, prevalence, risk factors and challenges were included. The protocol for the review was registered with PROSPERO (registration number CRD42022311609). Pooled prevalence rates were calculated using the programme R version 4.2.1. Heterogeneity was assessed using the I2 statistic and p-value. A narrative approach was used to describe risk factors and challenges. Studies were selected and finalised based on the review question to prioritise. The quality of the included studies was assessed using the Mixed-Method Appraisal Tool (MMAT).

**Results:**

A total of 248 publications met the requirements for inclusion. Fifteen articles were included in this review, with the result showing high heterogeneity. The pooled prevalence of urinary schistosomiasis in children is 4% (95% confidence interval (CI)). Age, poor socioeconomic status, education, exposure to river water, and poor sanitation are the risk factors identified in this review. Challenges are faced due to limitations of clean water, lack of water resources, and poor hygiene.

**Conclusion:**

Modifiable risk factors such as poor knowledge and practices must be addressed immediately. Healthcare providers and schools could accomplish engaging in practical promotional activities. Communicating the intended messages to raise community awareness of urinary schistosomiasis is critical.

## Introduction

Schistosomiasis is a parasitic infection with serious public health consequences in tropical countries [1,2]. This disease has infected 240 million people and 133.3 million preschoolers. *Schistosoma haematobium* species is blamable for causing urinary schistosomiasis, which occurs in Africa, Turkey, and India [3].

Cercariae from Bulinus snails act as an intermediate host in infected water that penetrates the human skin. Adult schistosomes travel to the bladder’s vesicular and pelvic venous plexus then Females deposit eggs in the bladder and ureters. Some parasite eggs are expelled, while others might build up in bladder tissues, causing ulceration, hematuria, and UTI symptoms [4]. The life cycle of Schistosoma haematobium in freshwater is shown in S1 Fig.

The infected child may unknowingly be carrying the infection. The diagnosis is based on the detection of eggs under a microscope. Infection is also indicated by the presence of antibodies and/or antigens in blood or urine samples. The standard diagnostic technique is filtration using nylon, paper, or polycarbonate filters. Chemical reagent strips can detect microscopic blood in children’s urine if they exhibit hematuria [4]. Chronic active or chronic late urinary schistosomiasis is diagnosed using ultrasound. For mass screening, detecting worm antigens, DNA, and specific antibodies is prohibitively expensive [5]. If left untreated, chronic irritation in the bladder may result in iron deficiency anaemia, malnutrition, impaired development, and a higher risk for bladder cancer later in life [6].

The risk of infection is linked to demographic characteristics [such as age, sex, and occupation) and environmental factors [7]. The USC intermediate host snail prefers still or slow-moving bodies of water, such as lakes. Some residences lack bathrooms, and in Africa and Asia, open defecation is common, contaminating water bodies with infectious faeces [8]. As a result, the infection affects more underprivileged and poor rural groups, particularly women and people in agriculture and fishing areas [4,7].

Children are more susceptible to infection due to poor hygiene and exposure to contaminated water. The age of exposure to infected water is younger in high endemic areas and may become more frequent as age increases due to multiple visits [9]. The other important risk factors include parents and caregivers [7],who bring their child swimming, fishing, and animal bathing as an outdoor activity [10]. Although school children are taught to avoid contaminated water, the lack of choices drives their contact with infected water.

Other known barrier to eliminating schistosomiasis is a lack of safe water provided by the government [11]. Shortage of piped water supply forces communities to rely on freshwater sources for domestic, school, and recreational purposes. The school project initiative provides rainwater-harvesting tanks for all public schools in Kenya [12] as alternative, but the approach did not meet the whole purpose as it only works during the rainy season.

For prevention and controlling schistosomiasis, the World Health Organization (WHO) proposed praziquantel-based preventive chemotherapy given to school-aged children aged 5 to 15 [1]. Over the past few decades, schistosomiasis has been controlled chiefly through mass drug administration (MDA) of praziquantel (PZQ) [13]. However, PZQ has limitations against juvenile schistosomes and cannot prevent re-infection, causing schistosomiasis incidence and transmission to remain unsolved. No schistosomiasis vaccines are currently available [13].

The study on disease prevalence, risk factors and addressing the challenges are fundamental in understanding schistosomiasis. Subsequently, this systematic literature review aims to identify the current knowledge of USC on its epidemiology, risk factors, and challenge to help spread the knowledge in tackling the control of the infection and reducing significant complications of schistosomiasis.

## Methods

This review was registered with PROSPERO (registration number CRD42022311609). A literature search was conducted on PubMed, Web of Science and Scopus for studies published from January 2016 to November 2021. To identify and describe the up-to-date evidence of USC, date limitation was used. As a result, we chose the year 2016 as the beginning of the search. In addition, to look for unpublished theses, specific research centres and library sources have been searched.

We used the CoCoPop (Condition, Context, Population) [1] and PECO (Population, Exposure, Comparator, Outcome) research question concepts to determine the inclusion and exclusion criteria. Based on this concept, three main aspects were included: child participants (population), risk factors (exposure) and urinary schistosomiasis (outcome). The three aspects were then combined into one research question:

1. What is the prevalence/incidence (Co) of urinary schistosomiasis (Co) in children (P)?
2. What risk factors (E) increase the risk of urinary schistosomiasis (O) in children exposed to it (P)? [1]

Terms “trends” or “prevalence” or “epidemiology” “distribution” or “burden” AND “Schistosomiasis” OR “Schistosoma” AND “urinary” OR “bladder” AND “child” or “paediatric” or “kids” AND “risk factors” or “determinants” or “predictors” were used in various combinations as primary search keywords.

The accepted articles were based on the following criteria:

- Study populations that included children under 18 years of age, without restriction of sex or region
- Studies that addressed risk factors associated with urinary schistosomiasis
- Studies that investigated the prevalence and challenges of USC
- All accepted observational and intervention studies, including randomised, quasi-randomised and non-randomised.

All original, primary studies conducted on the epidemiology, risk factors and challenges of urinary schistosomiasis with all full-text English-language published between Jan 1, 2016, to 30 November 2021, qualified to be included in this systematic review.

We excluded articles that did not relate to USC, USC risk factors and prevalence or studies with non-human subjects, study protocols, conference abstracts, dissertations, reviews, qualitative studies, editorials, case studies and opinion articles. Studies with inadequate data on desired information were also excluded. All duplicate studies were deleted, and the full text of the papers was manually and electronically searched using databases.

### Screening and selection for inclusion

Firstly, the prescreen was done for study titles and abstracts against the eligibility criteria in phase one. The steps were performed by four reviewers (N.A.M., N.A., T.K. and RO). All reviewers screened for study titles and abstracts independently. Any unrelated articles were removed in this phase. The full texts of the studies identified as potentially relevant in the initial screening were retrieved and screened against the eligibility criteria. After that, four reviewers read the full text independently (N.A.M., N.A., T.K. and RO). Any uncertainty regarding study eligibility was discussed and resolved with another reviewer. Disagreements were resolved through discussion consensus among the five reviewers and input from a lead reviewer.As shown in S2 Fig., a PRISMA-based flow diagram is used to record and illustrate the study selection process at the different stages.

### Data extraction

The data from included studies were gathered and documented on a standardised data extraction form by two reviewers (NAM and NA) to document related items and entered in Microsoft Excel with the research information such as authors, year of publication, countries, study design, prevalence, risk factors and challenges. Another reviewer (RO and TK) double-checked the accuracy of the extracted data.

### Data synthesis

A narrative approach was used to describe data primarily. The results relevant to the review question were extracted and organised in data extraction form. The studies were chosen and concluded based on the review question in order to prioritise the results summary. This informal method was accomplished by organising the tables and figures used to investigate heterogeneity. The findings include the number of studies and participants, the risk of bias in the studies, the directness of the study addressing the review question and the risk of publication bias to address the certainty of the evidence. The data are presented in tables to compare the findings from included studies. Three independent reviewers (R.O., T.K. and N.A.) double-check the accuracy of the extracted data. To ensure consistency in the results section, all reviewers need to agree on a structure for reporting results.

### Data analysis

We performed an initial descriptive analysis of the studies. The I2 statistic was used to assess the heterogeneity of the studies. An I2 value of more than 75% indicates high heterogeneity. As the reported results were very diverse, not all included studies could be used for a meta-analysis. It is excluded if the reviewers conclude that one study is insufficient to add to the evidence base. Pool estimates for prevalence and their 95% confidence interval were obtained with R statistical software version 4.2.1 using the statistical package ’dosresmeta’ by Robert Gentleman and Ross Ihaka of the Department of Statistics, University of Auckland, New Zealand.

### Quality assessment of individual studies

The quality of included studies was assessed using Mixed-Method Appraisal Tool (MMAT) assessment [15]. Quality appraisal was conducted using the Mixed Method Appraisal Tool (MMAT). The MMAT evaluates the quality of qualitative, quantitative, and mixed-method studies. It focuses on methodological criteria and includes five core quality criteria for each of the following five categories of study designs: (1) quantitative, (2) qualitative, (3) randomised controlled (4) nonrandomised, and (5) mixed methods. Four reviewers (NAM, NA, TK and RO) independently assessed the quality of the included studies. The reviewers’ discrepancy was managed through discussion, and articles were included after consensus. The quality assessment tool measures a total of nine questions. The highest score from seven questions indicated a low risk of bias. Overall scores of 0–2, 3–5, and 6–7 was declared the low, moderate, and high risk of bias, respectively. The quality assessment is depicted in Table 1.

**Table 1:** The details of the Mixed-Method Appraisal Tool (MMAT) assessment.

	Screening questions		Quantitative Descriptive Studies					
First author	S1. Are there clear research questions?	S2. Do the collected data allow to address the research questions?	4.1. Is the sampling strategy relevant to addressing the research question?	4.2. Is the sample representative of the target population?	4.3. Are the measurements appropriate?	4.4. Is the risk of nonresponse bias low?	4.5. Is statistical analysis appropriate to answer the research question?	Score
Mutsaka-Makuvaza et al. (2019) [6]	Yes	Yes	Yes	Yes	Yes	Can’t tell	Yes	6
Moyo et al. 2016) [12]	Yes	Yes	Cannot tell	Cannot tell	Yes	Cannot tell	Yes	4
Sulieman et al. (2017) [16]	Yes	Yes	Cannot tell	Cannot tell	Cannot tell	Cannot tell	Yes	3
Kabuyaya et al. (2018) [18]	Yes	Yes	Yes	Yes	Yes	Cannot tell	Yes	6
Mewabo et al. (2017) [27]	Yes	Yes	Yes	Cannot tell	Yes	Cannot tell	Yes	6
Yauba et al. (2018) [17]	Yes	Yes	Yes	Yes	Yes	Cannot tell	Yes	6
Sumbele et al. (2021) [19]	Yes	Yes	Yes	Yes	Yes	No	Yes	6
Molvik et al. (2017) [25]	Yes	Yes	Yes	Yes	Yes	Cannot tell	Yes	6
Abdulkareem et al 2018 [22]	Yes	Yes	Yes	Cannot tell	Yes	Cannot tell	Yes	5
Angora et al. (2019) [24]	Yes	Yes	Yes	Yes	Yes	Yes	Yes	7
Chadeka et al. (2017) [13]	Yes	Yes	Yes	Yes	Yes	Yes	Yes	7
Kabuyaya et al. (2017) [36]	Yes	Yes	Yes	Yes	Yes	Yes	Yes	7

## Results

The search yielded 15 articles from PubMed, 108 from SCOPUS, and 86 from WOS, resulting in 202 unique hits. After rigorous selection screening, only 15 articles were included in the full-text assessment, as shown in the PRISMA flow diagram (S2 Fig). Table 2 present a descriptive summary of the included studies in this review. This systematic review included the findings of 15 studies, as shown in Table 3.

**Table 2:** Summary of study location and study design.

Study location	Authors (Year)
Nigeria	Abdulkareem et al. (2018) [22]; Yauba et al. (2018) [17]
Kenya	Chadeka et al. (2017) [13]
Malawi	Moyo et al. (2016) [12]
Cameroon	Mewabo et al. (2017) [27]; Sumbele et al. (2021) [19]
South Afrika	Angora et al. (2019) [24]; Kabuyaya et al. 2017 [36]; Kabuyaya, Chimbari & Mukaratirwa (2018) [18]; Molvik et al. (2017) [25]
Zimbabwe	Mutsaka-Makuvaza et al. (2019) [6]
Sudan	Sulieman et al. (2017) [16], Hajissa et al. (2018) [20]
Gambia	Joof et al. (2021) [21]
Mauritania	Gbalégba et al. (2017) [23]
**Study Design**	
Cross-sectional	Abdulkareem et al. (2018) [22], Yauba et al. (2018) [17] Chadeka et al. (2017) [13], Moyo et al. (2016) [12], Sumbele et al. (2021) [19], Mewabo et al. (2017) [27] Molvik et al. (2017) [25], Angora et al. (2019) [24] (Sulieman et al., 2017) [16] Mutsaka-Makuvaza et al. (2019) [6], Kabuyaya et al. (2017) [36], Joof et al. (2021) [21], Hajissa et al. (2018) [20], Gbalégba et al. (2017) [23]
Cohort study	Kabuyaya, Chimbari & Mukaratirwa (2018) [18]

**Table 3:** Summary of study location and study design.

No	Author (Year), Country	Study Design	Sample Size	Prevalence	Risk factors studied	OR/AOR	CI	P value	Conclusion	Challenges in controlling and eliminating USC
1.	Sumbele et al. (2021) [19], Cameroon	Cross-sectional	606 school-age children	16.3	Age Sex nutritional status			All are >0.05	No significant association	-
2.	Molvik et al. (2017) [25], South Africa	Cross-sectional	726 primary school girls	36.9	Presence of soil transmitted helminths (STH) infection	2.05	1.58–2.93;	p<0.001	A significant association was found between *S*. *haematobium* and STHs	-
					Red urine, dysuria, washing blankets in the river and swimming in the river	-	-	-	Presenting with symptoms such as red urine, dysuria, washing blankets in the river and swimming in the river were associated with *S*. *haematobium*	-
3.	Abdulkareem et al. (2018) [22], Nigeria	Cross-sectional	724 students (4–18 years old)	45.6	Significant Male Unemployment Frequent water contact Lack of portable water Lack of awareness	1.98 2.23 2.01 4.76 2.16	0.65–2.19 1.89–2.94 1.45–2.70 2.64–5.98 0.59–3.83	0.007 0.036 0.047 <0.001 0.001	Significant increased risk of US in male, unemployed, lack of awareness, frequent water contact and lack of portable water.	-
4.	Angora et al. (2019) [24], Africa	Cross-sectional	1187 schoolchildren (5–14 years old)	14.0	Swimming Playing in the water	127.0 74.0	25.0–634.0 3.8–144.3	- -	Swimming in open freshwater bodies was the main risk factor for *S*. *haematobium* infection	-
5.	Chadeka et al. (2017) [13], Kenya	Cross-sectional	368 school children from 6 primary school	33.2	SES (very poor vs least poor) Religion (Muslim)	Z value 2.31 3.26	-	0.0208 0.0011	High intensity of *S*. *haematobium* infection was associated with very poor and Muslim	-
6.	Kabuyaya et al. (2017) [36], South Africa	Cross-sectional	320 school-going children aged 10–15 years	37.5	Age (12 compared to 10) Distance less than one kilometre to open water Swimming in infested water	0.296 3.273 2.147	0.114–0.764 1.216–8.807 1.213–3.799		The risk factors associated with schistosomiasis were age, near to water source and swimming in infested water.	-
7.	Kabuyaya et al. (2018) [18], South Africa	Cohort	173 school going children	37.5	Demographic, sanitation, recreational and occupational activities			All are >0.05	No significant difference was found among the risk factors for schistosomiasis	-
8.	Mewabo et al. (2017) [27], Cameron	Cross-sectional	229 pupils	16.6	Four times per week to the river/dam	-	-	0.01	Prevalence of infection was significantly associated with frequent direct contact to river	-
9.	Yauba et al. (2018) [17], Nigeria	Cross-sectional	385 children	62.0	Stunting, Low social class	-	-	0.04 0.01	USC more frequently associated with stunting and low social class	Limited water sources
10.	Mutsaka-Makuvaza et al.et al. (2019) [6] Zimbabwe	Cross-sectional	860 children	15.4	Child to caregivers who use river water Infected caregivers	2.2 3.9	1.3–3.7 1.7–8.6		Children whose caregivers used river water for bathing and infected were more likely to be infected.	Limited safe water sources and lack of knowledge on prevention and control of the disease
11.	Moyo et al. (2016) [12], Malawi	Cross-sectional	143 preschools	13	4-year-old 5.26x higher risk than 2-year-old	5.255	1.014–27.237	0.048	age was statistically significantly associated with urinary schistosomiasis risk	Limited clean water sources Preventing the community from daily visit to infested sites
12.	Sulieman et al. (2017) [16], Sudan	Cross-sectional	385 children	1.82	-	-	-	-	-	Lack of information (about the causes and ways of preventing the infection)
13	Hajissa et al. (2018) [20] Sudan	Cross- sectional	170 aged 6 and 17 years from two selected primary schools	12.9	Aged between 10 and 13 years Participants who had non-sanitary latrine	-	-	-	No significant associated risk factors found for USC	-
14	Joof et al. (2021) [21], Gambia	Cross sectional	2,018 students between the ages of 7 and 14 years	10.2	Male Bathing, playing and swimming in water bodies	-	-	-	Bathing, playing and swimming were found to be less risk for *S*. *haematobium* infection	-
15	Gbalégba et al. (2017) [23], Mauritania	Cross sectional	2162 children aged 5–15 years	4.0	Male Primary school child Dry season	1.78 1.73 0.56	1.13–2.80 1.04–2.87 0.35–0.89	-	Being male, being at primary school and dry season were significantly associated with *S*. *haematobium*.	-

OR, Odd ratio; aOR, adjusted Odd Ratio.

Four articles were from South Africa: two articles from Cameroon, two articles from Nigeria and Sudan, and one article each from Kenya, Malawi, Mauritania, Gambia and Zimbabwe. The articles analysed were published between 2016 and 2021. Fourteen of the 15 articles were cross-sectional studies and one article was a cohort study.

### Prevalence of USC

The review found that the prevalence of urinary schistosomiasis among children ranged from 1.82% to 62.0%. The lowest prevalence was in Sudan[14] and the highest was 62% in Nigeria [15].

### Risk factors for USC

In four studies included in this review, age was linked to USC [11,14,16–18]. One study found that four-year-old children had a higher risk than two-year-old children [11], and a Sudanese article found that children aged 7 to 10 years old had a higher risk than children aged 10 to 14 years old [14]. Aside from that, four articles [17,19–21] identified gender, particularly boys, as a risk factor in the findings.

Low socioeconomic status and poor education also played significant roles. Children with low socioeconomic status and poor knowledge were more vulnerable to USC than children with higher socioeconomic status [12,15,20]. The child’s current health status, such as stunting, is an important risk factor as the children with stunting had a higher risk of this infection [15,17].

Aside from that, a few articles identified any history of swimming, bathing, lack of clean water, previous exposure to river water [5,19,22] and has non sanitary larine [18] as the contributing factors. Poor hygiene, the presence of other infections such as soil helminth infection [23] and hookworm [12] over a year were also linked to USC.

Seasonal factors such as drought and praziquantel treatment were protective factors and lessened the risk [24]. A relative temperature range of 22–28°C preferable for snails as intermediate hosts [25] will increase the risk of USC.

### Challenges in controlling and eliminating USC

Five studies looked at the difficulties of controlling and eliminating USC [5,11,14,16,25].

The most common challenges were a lack of knowledge and information about the disease and the transmission process [11,14]. Most articles in low income countries discuss the limitations of clean water, a lack of water resources, and poor hygiene as challenges in controlling USC [15,25].

All 15 studies have adequate data to perform meta-analysis on prevalence of urinary schistosomiasis. The meta-analysis on the risk factors of urinary schistosomiasis was not possible due to inadequate data. R program version 4.2.1 was used to conduct the analysis. In order to conduct the analysis, articles that reported the prevalence in decimals [1–11] are converted to the nearest whole number to prevent non-integer count error. Random effect model was used to calculate the combined prevalence of urinary schistosomiasis in children. The pooled prevalence of urinary schistosomiasis in children is 4% (95% CI) [2–7]. Heterogeneity was assessed by the I^2^ statistics versus p-value. A p-value of ≤ 0.05 and I^2^ ≥ 50% were considered high heterogeneity. The heterogeneity generated was high at 96% and the p-value is less than 0.01 that indicates the test is significant.

## Discussion

This study examined the prevalence, risk factors, and challenges in controlling and eliminating USC. Urinary schistosomiasis was prevalent in Sub-Saharan Africa and remained a significant public health concern. Nigeria has the highest schistosomiasis burden in Sub-Saharan Africa, with an estimated 29 million cases of infection [26].

The previous study used MetaXL’s inverse variance heterogeneity model to obtain pooled prevalence estimates (PPE), and this review shows higher PPE for S. haematobium (IVhet PPE: 15%, 95% CI: 6–25) than our review [27]. The low prevalence could be attributed to the fact that the risk group includes children from preschool to 18 years old, as opposed to the previous review, which only included preschool-aged children. Furthermore, schistosomiasis has been primarily controlled through the widespread administration of praziquantel (MDA).

Four studies [11,14,16,17] found that increasing age was associated with urinary schistosomiasis among children. Infection was found in older children aged two to five years, indicating that infection with urinary schistosomiasis occurs early in life because of exposure to contaminated water. School-aged children are more likely to engage in independent water-based play and assist with duties that require contact with water bodies due to their advanced physical and motor development. However, Ugbomoiko et al. observed that the prevalence and intensity of USC over 14 decreased with age [24].

This review identified males as a risk factor for urinary schistosomiasis [14,17,20,25]. Similarly, previous studies showed that boys were more likely to be infected with urinary schistosomiasis than females due to socio-cultural and behavioural factors in which boys are more likely to swim, fish, and play in bodies of freshwater [19,28,29]. Other water-contact activities such as farming, watering cattle and other domesticated animals may result in increased exposure among boys. On the other hand, girls are socially restricted from engaging in water-contact activities such as swimming and bathing. Girls tend to stay home and do housework with tap water, limiting their exposure to other water sources [19].

The occurrence of schistosomiasis was linked to socioeconomic factors. According to Abdulkareem et al., the parent’s unemployment status significantly predicts schistosomiasis [20]. The high unemployment rate in the study area directly indicates low income and poverty. Poverty is linked to several issues, such as poor housing and sanitation, lack of piped water and access to anti-helminths [24].

Poor education may increase the risk of urinary schistosomiasis [30]. This link is most likely interconnected with education influencing attitudes and behaviour in various circumstances. They are more likely to engage in activities that expose them to cercarial penetration, such as crossing a stream or river barefoot. Furthermore, parents’ high illiteracy and negligence, particularly among farmers, can lead to non-education of preventive measures for their children and influence transmission patterns [26]. Stunting has been shown to predict infection with *S*. *haematobium* [31]. Stunted children are thought to be more susceptible to parasite infection for various reasons, including a reduction in T-lymphocyte function, defects in antibody synthesis, impaired complement formation, and degeneration of the thymus and other lymphoid tissues.

Climate and ecology have a significant impact on schistosomiasis transmission. During a drought in Ndumo, Kabuyaya et al. reported that *S*. *haematobium* declined two years after praziquantel therapy. Ecological and seasonal factors may play a role in schistosomiasis re-infection after treatment. Seasonal/climatic and ecological conditions, such as low rainfall, resulted in continuous drought and the drying up of water bodies (dams) that could have been schistosomiasis hotspots in the study area. Furthermore, drought heatwaves may cause human population concentrations near reservoirs, canals, and rivers, increasing contact with contaminated waterways and the risk of schistosomiasis transmission [16]. The tropical ecology of relative temperatures ranging from 22 to 28°C favours the development of intermediate snail hosts, which will increase the risk of infection [21]. Schistosome intermediate hosts are poikilotherms, which means that their body temperature fluctuates. Temperature influences reproduction, survival, dissemination, and parasite development within the snail. Climate change may alter the distribution and abundance of the intermediate host snail and its schistosome parasites, thereby altering disease dynamics and transmission to humans [32].

The relationship between schistosomiasis and freshwater interaction has long been established as schistosomiasis is known to infect humans during water contact behaviours. This review showed that swimming, bathing, and exposure to river water were contributing factors [14]. Sick children excreted *S*. *haematobium* eggs in their urine while swimming and later produced furcocercariae that penetrated children’s skin exposed to the contaminated water. Similar findings were observed in a study conducted in Senegal, where variability and epidemiology of the disease were attributable to water-contact patterns [29].

Water-contact behaviour could be linked with occupation, socioeconomic and socio-cultural factors. A study by Ugbomoiko et al. reported that water contact is recreational and domestic [33]. The father’s occupation as a farmer was significantly associated with urinary schistosomiasis. Children participated in field activities with their fathers [24], demonstrating a lack of awareness among fathers about the risk of urinary schistosomiasis and failing to inform their children about its risk. As a result, the protective role of the family’s head of household being literate and knowledgeable about urinary schistosomiasis is critical in preventing transmission. Fishers and farmers have been exposed to such infestations because their jobs require frequent contact with unknown risk water, necessitating the immediate implementation of community-based schistosomiasis risk factor surveillance and targeted interventions.[25].

Out of 15 articles included in this review, the most common challenges are associated with a limited source of clean water [34]. Limited clean water sources place the community at a higher risk of infection with urinary schistosomiasis. The role of clean water in infectious diseases has always been an essential factor that has been highlighted globally. For example, in 2016, United Nations Children’s Fund (UNICEF 2016) developed a strategy for water, sanitation and hygiene (WASH) to achieve Sustainable Development Goal (SDG) 6 by 2030. One of the reasons for the formation of WASH was the strong association between poor WASH and neglected tropical diseases such as guinea worm, schistosomiasis, helminths, and trachoma that lead to undesired outcomes in the health of the children. Access to safe drinking water has been linked to a significant reduction in STH infection. A study in Côte d’Ivoire discovered that drinking tap water at home is associated with a lower risk of *S*. *haematobium*, hookworm and *E*. *coli* [35].

Apart from that, poor knowledge is also a barrier to controlling and eliminating USC. The baseline study revealed that the intensity of USC was high, corresponding with the lack of knowledge [36]. After two years, knowledge of urinary schistosomiasis had improved, from 56.6% to 93% of participants knowledgeable about urinary schistosomiasis, and the intensity of infection had decreased [16]. It was noted that the school children acquired information on schistosomiasis from the school and parents. Schools and parents play a significant role in preventing and controlling USC. Claude et al. [37] found that local children and adults in Gabon have a good understanding of urinary schistosomiasis, but 60% of the community practices risk-enhancing behaviour, notably in the area that lacks piped water. The issue shows that knowledge and proper water facilities must be in tandem to control and eliminate schistosomiasis.

Besides the limitation of clean water sources and poor knowledge, community practices are also found to be a challenge in the effort to control and eliminate urinary schistosomiasis. In the study conducted by Moyo et al. [11], it was found that tricky to prevent the community from visiting the infested sites for daily activities. Similar findings are also portrayed in the study conducted [37] involving 602 study participants. Out of the 602 study participants, 387(64%) participants reported urinating in a freshwater body and 152 (25%) participants regularly defecated in a freshwater body.

This review had limitations as most publications focused on assessing and treating primary school children. Other vulnerable populations, such as preschool children and children under six, may be at risk of underreporting. As non-English papers were not included in this review, language bias was possible. We overlooked findings from the grey literature because we limited our review to three databases and freely available, published, peer-reviewed full texts.

Several systematic reviews of USC have been conducted, but our review may be unique in that it focuses on recent meta-analyses of prevalence, risk factors in children, and challenges in preventing USC, while other papers may focus on other risk categories. Few studies, such as this one, highlight the difficulties in preventing and controlling the infection.

## Conclusion and recommendations

Modifiable risk factors such as poor knowledge and practices need to address immediately. This could be achieved through continuous practical promotional activities by the relevant stakeholders such as the health care provider and school. It is crucial to ensure that intended messages are delivered to increase community awareness of urinary schistosomiasis.

In this review, USC remains a neglected tropical disease that needs further improvement despite various efforts implemented globally. The disparity in the incidence of urinary schistosomiasis between developed and underdeveloped countries is salient. It highlights those necessities, such as clean and adequate water supply, hinder the reduction of urinary schistosomiasis.

Given the negative impact of urinary schistosomiasis, a holistic approach involving the stakeholders such as parents, schools, and the government is needed to control and eliminate urinary schistosomiasis. Apart from that, a multisectoral partnership involving non-governmental organisations (NGOs) and the government is needed to ensure a clean and adequate water supply, as it has an essential role in reducing urinary schistosomiasis in the community.

Further research and development are needed, especially in underdeveloped countries. For example, a non-sewered sanitation system that uses nanomembrane technology could be helpful, especially in a limited water supply [38]. Besides that, policymakers should ensure adequate financing to build the appropriate infrastructure for the water supply.

## Supporting information

S1 Checklist Appendix. Preferred Reporting Items for Systematic Reviews and Meta-Analyses (PRISMA) checklist.
(DOCX)

S1 Fig Life cycle of *Schistosoma haematobium* in freshwater. (TIF)

S2 Fig The study selection process. (TIF)

S3 Fig Meta-analysis on urinary schistosomiasis in children. (TIF)

## References

[1] Deol AK, Fleming FM, Calvo-Urbano B, Walker M, Bucumi V, Gnandou I, et al. Schistosomiasis—Assessing Progress toward the 2020 and 2025 Global Goals. N Engl J Med [Internet]. 2019 Dec 26 [cited 2022 Jan 9];381(26):2519–28. Available from: https://www.nejm.org/doi/full/10.1056/NEJMoa1812165. 31881138 10.1056/NEJMoa1812165 PMC6785807

[2] World Health Organization. Schistosomiasis and soil-transmitted helminthiases: progress report, 2020 [Internet]. Geneva; 2021 [cited 2022 Jan 6]. Available from: https://apps.who.int/iris/handle/10665/350004?show=full.

[3] Chelsea M, William A. Petri J. Schistosomiasis—Infectious Diseases—MSD Manual Professional Edition [Internet]. Merck Sharp & Dohme Corp. 2021 [cited 2022 Jan 6]. Available from: https://www.msdmanuals.com/professional/infectious-diseases/trematodes-flukes/schistosomiasis.

[4] CDC. Schistosomiasis [Internet]. Division of Parasitic Diseases and Malaria CDC. 2019 [cited 2022 Jan 9]. Available from: https://www.cdc.gov/dpdx/schistosomiasis/index.html.

[5] WHO. Schistosomiasis [Internet]. WHO. 2021 [cited 2022 Jan 2]. Available from: https://www.who.int/news-room/fact-sheets/detail/schistosomiasis.

[6] Mutsaka-Makuvaza MJ, Matsena-Zingoni Z, Katsidzira A, Tshuma C, Chin’Ombe N, Zhou XN, et al. Urogenital schistosomiasis and risk factors of infection in mothers and preschool children in an endemic district in Zimbabwe. Parasites and Vectors [Internet]. 2019;12(1):1–15. Available from: 10.1186/s13071-019-3667-5. 31477172 PMC6721289

[7] Gaber DA, Wassef RM, El-Ayat WM, El-Moazen MI, Montasser KA, Swar SA, et al. Role of a schistosoma haematobium specific microRNA as a predictive and prognostic tool for bilharzial bladder cancer in Egypt. Sci Reports 2020 101 [Internet]. 2020 Nov 2 [cited 2022 Jan 6];10(1):1–10. Available from: https://www.nature.com/articles/s41598-020-74807-1. doi: 10.1038/s41598-020-74807-1 33139749 PMC7606480

[8] Sacolo-Gwebu H, Chimbari M, Kalinda C. Prevalence and risk factors of schistosomiasis and soil-transmitted helminthiases among preschool aged children (1–5 years) in rural KwaZulu-Natal, South Africa: A cross-sectional study. Infect Dis Poverty [Internet]. 2019 Jun 16 [cited 2022 Jan 2];8(1):1–12. Available from: https://idpjournal.biomedcentral.com/articles/10.1186/s40249-019-0561-5. 31202273 10.1186/s40249-019-0561-5 PMC6571117

[9] Gordon CA, Kurscheid J, Williams GM, Clements ACA, Li Y, Zhou XN, et al. Asian Schistosomiasis: Current Status and Prospects for Control Leading to Elimination. Trop Med Infect Dis [Internet]. 2019 Feb 26 [cited 2022 Jan 9];4(1). Available from: /pmc/articles/PMC6473711/. doi: 10.3390/tropicalmed4010040 30813615 PMC6473711

[10] Osakunor DNM, Woolhouse MEJ, Mutapi F. Paediatric schistosomiasis: What we know and what we need to know. PLoS Negl Trop Dis [Internet]. 2018 Feb 8 [cited 2022 Jan 2];12(2). Available from: /pmc/articles/PMC5805162/. doi: 10.1371/journal.pntd.0006144 29420537 PMC5805162

[11] Favre TC, Massara CL, Beck LCNH, Cabello RKSA, Pieri OS. Adherence to diagnosis followed by selective treatment of schistosomiasis mansoni and related knowledge among schoolchildren in an endemic area of Minas Gerais, Brazil, prior to and after the implementation of educational actions. Parasite Epidemiol Control. 2021 May 1;13:e00208. doi: 10.1016/j.parepi.2021.e00208 33732914 PMC7941185

[12] Moyo VB, Changadeya W, Chiotha S, Sikawa D. Urinary schistosomiasis among preschool children in Malengachanzi, Nkhotakota District, Malawi: Prevalence and risk factors. Malawi Med J. 2016;28(1):10–4. doi: 10.4314/mmj.v28i1.3 27217911 PMC4864386

[13] Chadeka EA, Nagi S, Sunahara T, Cheruiyot NB, Bahati F, Ozeki Y, et al. Spatial distribution and risk factors of Schistosoma haematobium and hookworm infections among schoolchildren in Kwale, Kenya. PLoS Negl Trop Dis. 2017;11(9):1–17. doi: 10.1371/journal.pntd.0005872 28863133 PMC5599053

[14] Molehin AJ. Schistosomiasis vaccine development: Update on human clinical trials. J Biomed Sci [Internet]. 2020 Jan 22 [cited 2022 Jan 9];27(1):1–7. Available from: https://jbiomedsci.biomedcentral.com/articles/10.1186/s12929-020-0621-y. 31969170 10.1186/s12929-020-0621-y PMC6977295

[15] Munn Z, MClinSc SM, Lisy K, Riitano D, Tufanaru C. Methodological guidance for systematic reviews of observational epidemiological studies reporting prevalence and cumulative incidence data. Int J Evid Based Healthc [Internet]. 2015 Sep 1 [cited 2022 May 7];13(3):147–53. Available from: https://journals.lww.com/ijebh/Fulltext/2015/09000/Methodological_guidance_for_systematic_reviews_of.6.aspx. doi: 10.1097/XEB.0000000000000054 26317388

[16] Sulieman Y, Eltayeb RE, Pengsakul T, Afifi A, Zakaria MA. Epidemiology of urinary schistosomiasis among school children in the alsaial Alsagair village, River nile state, Sudan. Iran J Parasitol. 2017;12(2):284–91. 28761490 PMC5527040

[17] Yauba SM, Rabasa AI, Farouk AG, Abdullahi H, Ummate I, Ibrahim BA, et al. of Kidney Diseases and Transplantation Renal Data from Asia–Africa Urinary Schistosomiasis in Boko Haram-related Internally Displaced Nigerian Children. 2018;29(6):1395–402. 10.4103/1319-2442.248286 30588972

[18] Kabuyaya M, Chimbari MJ, Mukaratirwa S. Infection status and risk factors associated with urinary schistosomiasis among school-going children in the Ndumo area of uMkhanyakude District in KwaZulu-Natal, South Africa two years post-treatment. Int J Infect Dis [Internet]. 2018;71:100–6. Available from: doi: 10.1016/j.ijid.2018.04.002 29679769

[19] Sumbele IUN, Otia OV, Francis L, Bopda OSM, Ebai CB, Ning TR, et al. Confounding influences of malnutrition and Plasmodium falciparum and Schistosoma haematobium infections on haematological parameters in school children in Muyuka, Cameroon. BMC Infect Dis. 2021;21(1):1–13. 34034666 10.1186/s12879-021-06201-9 PMC8152139

[20] Hajissa K, Muhajir AEMA, Eshag HA, Alfadel A, Nahied E, Dahab R, et al. Prevalence of schistosomiasis and associated risk factors among school children in Um-Asher Area, Khartoum, Sudan. BMC Res Notes [Internet]. 2018;11(1):1–5. Available from: 10.1186/s13104-018-3871-y. 30382901 PMC6211415

[21] Joof E, Sanyang AM, Camara Y, Seyid AP, Baldeh I, Jah SL, et al. Prevalence and risk factors of schistosomiasis among primary school children in four selected regions of the gambia. PLoS Negl Trop Dis. 2021;15(5):1–15. doi: 10.1371/journal.pntd.0009380 33974623 PMC8139473

[22] Abdulkareem BO, Habeeb KO, Kazeem A, Adam AO, Samuel UU. Urogenital Schistosomiasis among Schoolchildren and the Associated Risk Factors in Selected Rural Communities of Kwara State, Nigeria. J Trop Med. 2018;2018. doi: 10.1155/2018/6913918 29853921 PMC5954937

[23] Gbalégba NGC, Silué KD, Ba O, Ba H, Tian-Bi NTY, Yapi GY, et al. Prevalence and seasonal transmission of Schistosoma haematobium infection among school-aged children in Kaedi town, southern Mauritania. Parasites and Vectors. 2017;10(1):1–12. 28747222 10.1186/s13071-017-2284-4 PMC5530530

[24] Angora EK, Menan H, Rey O, Tuo K, Tour AO, Coulibaly JT, et al. Prevalence and Risk Factors for Schistosomiasis among Schoolchildren in two Settings of C ô te d ‘ Ivoire. 2019. 10.3390/tropicalmed4030110 PMC6789509 31340504

[25] Molvik M, Helland E, Zulu SG, Kleppa E, Lillebo K, Gundersen SG, et al. Co-infection with Schistosoma haematobium and soil-transmitted helminths in rural South Africa. S Afr J Sci. 2017;113(3–4):2–7.

[26] Ugbomoiko US, Ofoezie IE, Okoye IC, Heukelbach J. Factors associated with urinary schistosomiasis in two peri-urban communities in south-western Nigeria. Ann Trop Med Parasitol. 2010;104(5):409–19. doi: 10.1179/136485910X12743554760469 20819309

[27] Mewabo AP, Moyou RS, Kouemeni LE, Ngogang JY, Kaptue L, Tambo E. Assessing the prevalence of urogenital schistosomaisis and transmission risk factors amongst school-aged children around Mapé dam ecological suburbs in Malantouen district, Cameroon. Infect Dis Poverty. 2017;6(1):6–13. 28260525 10.1186/s40249-017-0257-7 PMC5338087

[28] Amuta EU, Houmsou RS. Prevalence, intensity of infection and risk factors of urinary schistosomiasis in pre-school and school aged children in Guma Local Government Area, Nigeria. Asian Pac J Trop Med. 2014;7(1):34–9. doi: 10.1016/S1995-7645(13)60188-1 24418080

[29] Geleta S, Alemu A, Getie S, Mekonnen Z, Erko B. Prevalence of urinary schistosomiasis and associated risk factors among Abobo Primary School children in Gambella Regional State, southwestern Ethiopia: A cross sectional study. Parasites and Vectors. 2015;8(1):1–9. doi: 10.1186/s13071-015-0822-5 25886292 PMC4399218

[30] Senghor B, Aldioma D, Seydou N S, Souleymane D, Mamadou O. N, Lobna G, et al. Prevalence and intensity of urinary schistosomiasis among school children in the district of Niakhar, region of Fatick, Senegal. Parasites and Vectors. 2014;7(1):no pagination. 10.1186/1756-3305-7-5 PMC3882112 24387599

[31] Ayeh-Kumi PF, Addo-Osafo K, Attah SK, Tetteh-Quarcoo PB, Obeng-Nkrumah N, Awuah-Mensah G, et al. Malaria, helminths and malnutrition: A cross-sectional survey of school children in the South-Tongu district of Ghana. BMC Res Notes. 2016;9(1):1–12. doi: 10.1186/s13104-016-2025-3 27118136 PMC4847346

[32] De Leo GA, Stensgaard AS, Sokolow SH, N’Goran EK, Chamberlin AJ, Yang GJ, et al. Schistosomiasis and climate change. BMJ. 2020;371(November):1–8.

[33] Houmsou RS, Agere H, Wama BE, Bingbeng JB, Amuta EU, Kela SL. Urinary schistosomiasis among children in Murbai and Surbai communities of Ardo-Kola Local Government Area, Taraba State, Nigeria. J Trop Med. 2016;2016. doi: 10.1155/2016/9831265 28096819 PMC5206853

[34] Mohammed Yauba S, Ibrahim Rabasa A, Garba Farouk A, Abdullahi Elechi H, Ummate I, Abdullahi Ibrahim B, et al. Renal Data from Asia-Africa Urinary Schistosomiasis in Boko Haram-related Internally Displaced Nigerian Children. Saudi J Kidney Dis Transpl [Internet]. 2018;29(6):1395–402. Available from: http://www.sjkdt.org. 30588972 10.4103/1319-2442.248286

[35] Coulibaly G, Ouattara M, Dongo K, Hürlimann E, Bassa FK, Koné N, et al. Epidemiology of intestinal parasite infections in three departments of south-central Côte d’Ivoire before the implementation of a cluster-randomised trial. Parasite Epidemiol Control. 2018;3(2):63–76. 29774300 10.1016/j.parepi.2018.02.003 PMC5952672

[36] Kabuyaya M, Chimbari MJ, Manyangadze T, Mukaratirwa S. Schistosomiasis risk factors based on the infection status among school-going children in the Ndumo area, uMkhanyakude district, South Africa. South African J Infect Dis [Internet]. 2017;32(2):67–72. Available from: 10.1080/23120053.2016.1266139.

[37] Claude J, Agobé D, Zinsou JF, Honkpehedji YJ, Edoa JR, Adegbité BR, et al. Knowledge, attitudes and practices pertaining to urogenital schistosomiasis in Lambaréné and surrounding areas, Gabon. Parasit Vectors. 2021;1–12. 34551819 10.1186/s13071-021-04905-0 PMC8456596

[38] Hennigs J, Ravndal KT, Parker A, Collins M, Jiang Y, Kolios AJ, et al. Science of the Total Environment Faeces–Urine separation via settling and displacement: Prototype tests for a novel non-sewered sanitation system. Sci Total Environ. 2021;753:141881. 32896734 10.1016/j.scitotenv.2020.141881 PMC7674630

