# Supplementary figures and images for: Systematic review with meta-analysis: Prevalence, risk factors, and challenges for urinary schistosomiasis in children (USC)
Source: PLoS One. 2023 Aug 17;18(8):e0285533. doi: 10.1371/journal.pone.0285533 (PMC10434872; doi:10.1371/journal.pone.0285533)

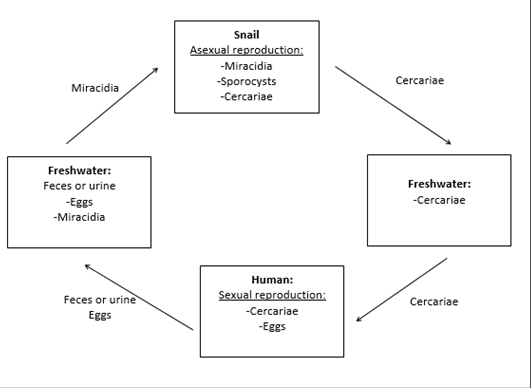

Supplement: S1 Fig — (TIF) [file pone.0285533.s002.tif]

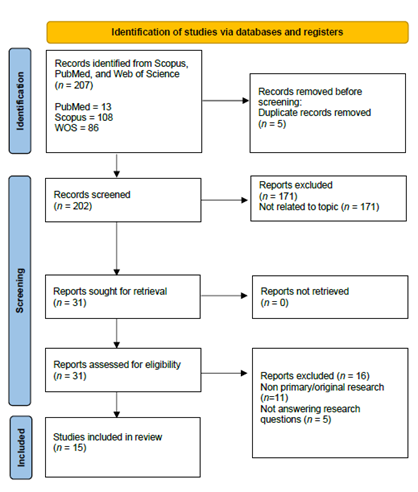

Supplement: S2 Fig — (TIF) [file pone.0285533.s003.tif]

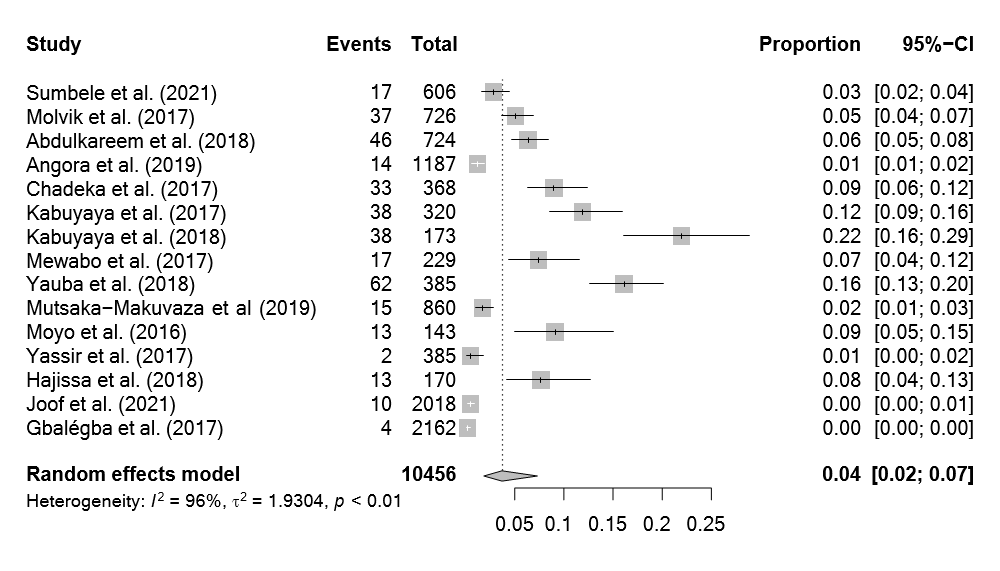

Supplement: S3 Fig — (TIF) [file pone.0285533.s004.tif]
